# Reviewed article: Filho JDS, Pinheiro MCC, Júnior ANR, Silva BM, Ferreira AF, Andrade TIB, Lacerda JM, Araújo LP, Belmino ACC, Oliveira MF, Bezerra FSM. Prevalence of neglected tropical diseases in rural productive villages of the São Francisco River Integration Project in Ceará: cross-sectional study, 2020. Epidemiol Serv Saude. 2025:34;e20240393.

**DOI:** 10.1590/S2237-96222025v34e20240393.a

**Published:** 2025-05-12

**Authors:** Giulia Vaz da Silva

**Affiliations:** FOUSP, Saúde Coletiva

**Table:** 

Rating	Excellent	Good	Average	Below average	Poor
Interest		✓			
Quality			✓		
Originality		✓			
Overall		✓			

**Table:** 

Questionnaire	Yes	No	Not applicable
Does the manuscript contain new and significant information to justify publication?	✓		
Does the Abstract (Summary) clearly and accurately describe the content of the article?	✓		
Is the problem significant and concisely stated?	✓		
Are the methods described comprehensively?		✓	
Are the interpretations and conclusions justified by the results?		✓	
Is adequate reference made to other work in the field?	✓		

## Manuscript Structure:

Length of article is: Adequate

Number of tables is: Too little

Number of figures is: Too little

Please state any conflict(s) of interest that you have in relation to the review of this paper (state “none” if this is not applicable): None

## Comments

Após uma análise cuidadosa do artigo, gostaria de compartilhar algumas considerações que, acredito, poderão contribuir para o aprimoramento do trabalho. Primeiramente, é fundamental revisar a ortografia e gramática do texto para garantir a clareza da comunicação. Além disso, sugiro a padronização das casas decimais para manter a consistência nos dados apresentados. Em relação ao uso do kit POC-CCA®, seria importante esclarecer o que significa a escala de Score G, incluindo suas referências e relevância no contexto do estudo. Também é necessário justificar a exclusão dos resultados G2 e G3 como positivos, apresentando os motivos que levaram a essa decisão.

A Tabela 1 pode necessitar de uma revisão para melhorar a clareza dos dados, incluindo uma legenda e uma reformulação na apresentação. Recomendo que o título da Tabela 2 especifique claramente a doença a que se referem os dados, em congruência com a Tabela 1. No terceiro parágrafo da Discussão, identifiquei uma afirmação especulativa que não é sustentada por dados apresentados no estudo e carece de referências bibliográficas que a corroboram, o que parece estar em desacordo com a literatura existente. Além disso, o oitavo parágrafo da Discussão contém uma afirmação que não se encontra nos resultados apresentados.

Sugiro também repensar os objetivos da pesquisa para garantir um alinhamento mais claro com a discussão. A inclusão de mais detalhes e informações que corroborem com as discussões feitas é essencial, pois, apesar dos métodos serem compatíveis com a pergunta de pesquisa, pode haver uma desconexão entre os objetivos do estudo e os dados apresentados.

Por fim, considero que uma melhor explicação sobre os métodos utilizados, especialmente em relação ao kit POC-CCA®, é necessária para aumentar a confiabilidade dos dados e facilitar o entendimento dos resultados. Agradeço pela oportunidade de revisar o artigo e espero que essas sugestões sejam úteis para o seu aprimoramento.

